# A Transforaminal Endoscopic Surgical Technique for Treating Lumbar Disc Herniation in the Setting of Spina Bifida

**DOI:** 10.1155/2020/1076847

**Published:** 2020-03-09

**Authors:** Albert E. Telfeian, Adetokunbo Oyelese, Jared Fridley, Rohaid Ali, Deus Cielo, Ziya L. Gokaslan

**Affiliations:** Department of Neurosurgery, Warren Alpert Medical School of Brown University, Providence, RI, USA

## Abstract

Recent literature suggests that adult patients with spina bifida receive surgery for degenerative disc disease at higher rates than the general population. However, sometimes the complex anatomic features of co-occurring spina bifida and lumbar disc herniation can significantly challenge standard surgical techniques. Here, the technical steps are presented for treating a foraminal lumbar 4-5-disc herniation in the setting of a patient with multifaceted degenerative and spina bifida occulta anatomy. Utilized is a minimally invasive approach that does not require general anesthesia or fusion and allows the patient to leave the same day. To the best of our knowledge, this is the first-reported case of endoscopic surgical decompression of a lumbar disc in a patient with spina bifida.

## 1. Introduction

For over fifty years, neurosurgeons have recognized that lumbar central stenosis and lateral recess stenosis can precipitate neurologic deterioration in adult patients with congenital neural tube defects [1, 2]. In fact, recent data demonstrate that patients with spina bifida receive operations for degenerative disc disease at higher rates than the general population [3]. Given this information, it is presumed that the U.S. population's burden of disease from co-occurring lumbar disc herniation and spina bifida is not insignificant, as the prevalence of spina bifida alone is estimated to be between 10 and 20% [4, 5]. Unfortunately, literature about the surgical management of lumbar disc herniation in the adult patient with spina bifida remains strikingly sparse [6–9].

It is within this context that we present a technical note for how to surgically treat a lateral disc herniation in an adult patient with spina bifida occulta (SBO) via a minimally invasive endoscopic approach. Over the past 40 years, techniques in endoscopic spine surgery have grown to effectively address an increasingly complex range of disorders, including tethered cord syndrome, pediatric spine tumor, and lumbar Tarlov cyst [10–13]. We deemed that our patient's part-degenerative, part-dysraphic anatomy meant that traditional, nonendoscopic surgical approaches would expose her to unacceptable clinical risk (e.g., CSF leak, direct nerve root injury, etc.), be technically infeasible, or introduce biomechanical instability necessitating fusion. Thus, we detail how our minimally invasive, awake (i.e., moderate conscious sedation), transforaminal endoscopic surgery was able to safely access and decompress the patient's lumbar nerve root without destabilizing her spine or requiring fusion.

## 2. Case Report

### 2.1. History and Presentation

**Figure 1:**
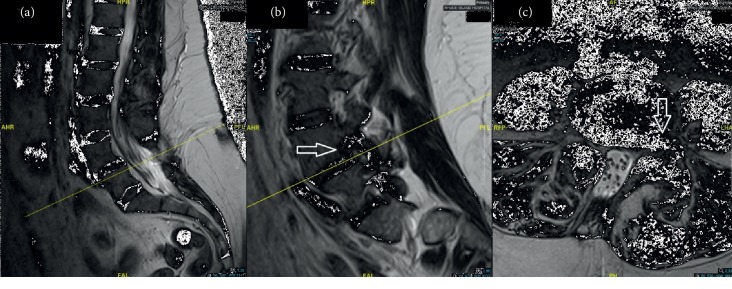
Preoperative lumbar MRI. (a) Midline sagittal T2-weighted MRI demonstrates the L4-5 spondylolisthesis and the prolapsed thecal sac. (b) Left of midline sagittal T2-weighted MRI with foraminal view demonstrates the left L4-5 (arrow) foraminal compression of the exiting L4 nerve. (c) Axial T2-weighted MRI demonstrates left L4-5 far lateral disc herniation compressing the exiting L4 nerve (arrow).

**Figure 2:**
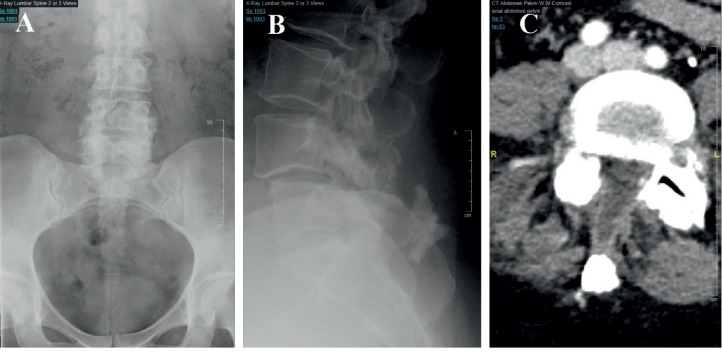
Preoperative X-ray and CT scan. (a) AP X-ray demonstrates the spina bifida occulta at L4-5; note the aberrant lamina. (b) Lateral X-ray demonstrates the L4-5 spondylolisthesis and the posterior subcutaneous calcification. (c) Axial CT image demonstrates the left L4-5 abnormal facet complex and the subcutaneous calcification.

A 51-year-old female presented after a motor vehicle accident with symptoms of a left L4 radiculopathy. On examination, she had left anterior thigh numbness and an absent left quadricep reflex. Over her lower lumbar area, she had a fat pad, asymmetric cleft, and hairy patch. She had already exhausted conservative treatment that included physical therapy and interventional pain management. An MRI of the lumbar spine (Figure 1) was performed and demonstrated a degenerative anterolisthesis and left lumbar 4-5 foraminal disc herniation. Also at the L4 level was a congenitally absent right L4 lamina through which the thecal sac had partially prolapsed. The conus medullaris was seen at the upper L2 level. A flexion-extension lumbar X-ray series demonstrated a Meyerding grade 1 anterolisthesis of L4 on L5 with no evidence of instability. Calcification in the posterior soft tissues at the level of L5 was seen on X-ray (Figures 2(a) and 2(b)) and CT (Figure 2(c)), likely related to spina bifida occulta [14]. An enlarged and dysmorphic left L4-5 facet complex can be appreciated on the CT, as well (Figure 2(c)).

After discussion of relevant possible interventions (see discussion for further details), the patient elected to undergo an awake, transforaminal lumbar endoscopic discectomy at the left L4-5 level.

### 2.2. Operative Procedure

**Figure 3:**
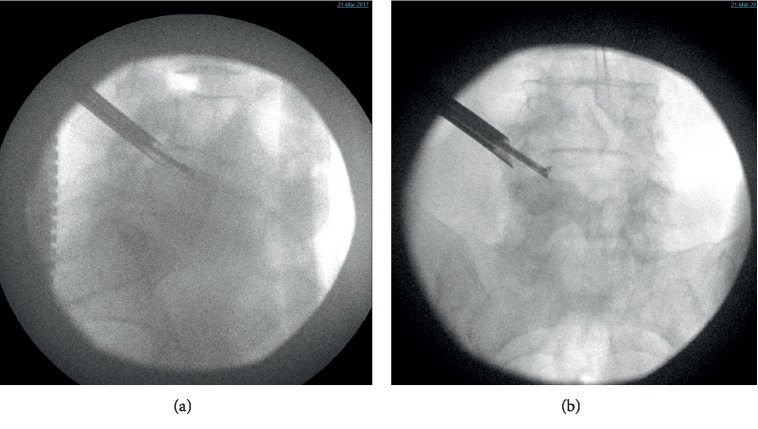
Intraoperative fluoroscopy. (a) Lateral fluoroscopic image of the beveled tubular retractor and endoscope at the L4-5 foramen. A semibendable grasper is displayed removing disc. (b) AP fluoroscopic image of the beveled tubular retractor and endoscope in the foramen and the rigid endoscopic grasper removing herniated disc.

**Figure 4:**
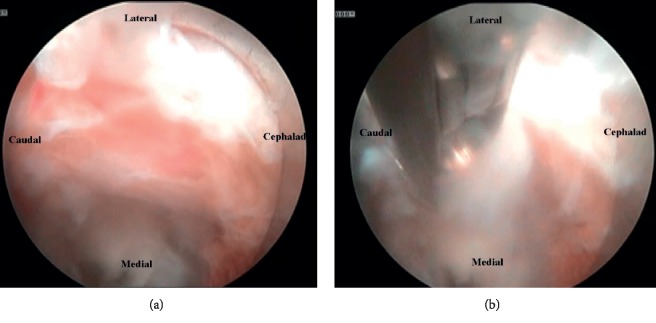
Endoscopic camera views of surgical procedure for the left lumbar 4-5 discectomy. (a) The 30-degree endoscopic camera is in Kambin's triangle upside down facing the L4 exiting root. The L4 root is demonstrated in this photo with a disc fragment seen below. (b) The endoscopic grasper is shown reaching under the L4 nerve root and removing the large disc fragment.

**Figure 5:**
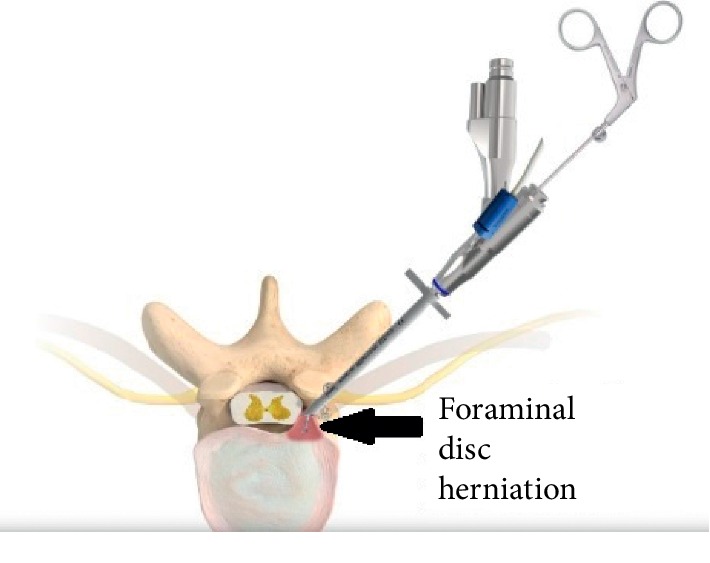
Schematic illustration of the transforaminal endoscopic surgical approach and instruments for a foraminal disc herniation.

For the endoscopic (Joimax TESSYS) left lumbar 4-5 discectomy procedure, the patient was positioned in the prone position on a Wilson frame with flexed hips and knees. The procedure was performed under local anesthesia (1% lidocaine with epinephrine) and intravenous sedation (midazolam and fentanyl); the level of anesthetic was titrated, so the patient was able to communicate with the surgeon throughout the procedure. Percutaneous entry was established through the skin 11 cm lateral to the midline. Using intermittent fluoroscopic guidance, alternating between lateral and anterior-posterior (AP) view, a 15 cm 18-gauge needle was advanced and placed at the superior endplate of the L5 vertebral body through Kambin's triangle, between the exiting and traversing nerves. The technique for placing the needle involved targeting the ventral edge of the superior articulating process where it meets the inferior pedicle. An AP fluoroscopic view was used to confirm the needle was at the medial border of the pedicle of L5. A 5 mm incision was made over the needle, and a K-wire was placed in the needle. The needle was removed, and sequential dilators were placed over the K-wire. Sequential reamers were used to enlarge the neural foramen by removing the ventral aspect of the superior articulating process of L5. At this point, the beveled cannula tubular dilator was placed over the sequential dilators, the dilators were removed, and the 7 mm outer diameter Joimax® rigid working channel endoscope channel was inserted through the tubular retractor.  Figure 3 demonstrates the position of the tubular retractor on AP and lateral fluoroscopy. Under endoscopic visualization, the L4 nerve root was directly visualized with the disc herniation compressing it from below (Figure 4(a)). An endoscopic grasper was used to reach under the nerve under fluoroscopic (Figure 3) and endoscopic visualization (Figure 4(b)) and remove the disc herniation.  Figure 5 is an illustration of the surgical approach and instruments. The patient was able to communicate during the procedure that her pain was immediately improved.

### 2.3. Postoperative Course

The postoperative course was uneventful, and the patient's left anterior thigh pain improved immediately after the surgery. The patient was discharged to home on the same day of her surgery. Six weeks, six months, and 2 years after her endoscopic procedure, the patient had no clinical symptoms referable to the disc herniation.

## 3. Discussion

Identifying safe and effective techniques to treat lumbar disc herniation is an important mission in the landscape of care for adults with spina bifida. As mentioned, these patients receive surgery for lumbar stenosis at higher rates than the general population, and as their mean life expectancy continues to grow, likely so will their prevalence of degenerative disc disease [3, 15]. The surgical challenges include safely traversing or, better, circumventing dorsal, dysraphic congenital pathology, which may also contain scarred tissue from previous spina bifida surgery [16]. Doing so in a manner that is simultaneously cost- and time-effective is an added bonus in recognition of the lifetime healthcare-related expenses and morbidity that many with spina bifida must endure [17]. In the case presented above, we demonstrate how a transforaminal, endoscopic approach is in line with these goals. A surgical approach should always be tailored to the individual patient, but we hope that highlighting this case's technical nuances and unique achievements will be of use for patients with pathology across the spina bifida spectrum.

To start, it is important to note that no definitive causal link has been established between congenital spina bifida and the development of lumbar disc herniation. Theoretical explanations point to embryologic aberrancies (i.e., the shared mesodermal origin of bones and the nucleus pulposus), biomechanical stressors (e.g., the cumulative trauma induced by lifelong gait disturbances), or some mixture of both [14, 18]. Empirical evidence for these theories, though, is lacking. Indeed, there is literature to suggest spina bifida does not accelerate lifelong degenerative changes [19, 20]. The relatively higher incidence of surgery for degenerative disc disease in patients with spina bifida may simply be explained by these patients' more frequent neurosurgical surveillance, a form of observer bias [3]. The implication for our case is that we did not view the presence of spina bifida occulta as an independent justification for adding arthrodesis to nerve root decompression.

Our patient's challenging constellation of degenerative and congenital lumbar pathology forced us to seriously consider the benefits and disadvantages of all standard surgical approaches. Although a comprehensive listing of techniques to address the patient's severe left L4-5 foraminal stenosis would be beyond the scope of this technical note, it is worth considering common posterior and anterior (i.e., relative to the transverse process) approaches to help illustrate why a transforaminal endoscopic approach was ultimately chosen here.

In terms of posterior lumbar approaches, our patient's dysraphic anatomy poses concerns regarding safety and stability. The posterior surgical procedures we consider here include posterior lumbar interbody fusion (PLIF), transforaminal lumbar interbody fusion (TLIF), and paramedian “Wiltse” microdiscectomy. There exists controversy regarding posterolateral versus interbody fusion, but because of theoretically improved foraminal decompression, when discussing posterior fusion techniques for this patient, we limit our consideration to the interbody fusion approaches [21]. To begin, PLIF achieves access to the disc space via a midline posterior incision, dissection and retraction of the paramedian muscles, laminectomy, and finally medial retraction of the thecal sac. We feel its usage would be inadvisable in this scenario for a number of reasons. Of the lumbar interbody fusion techniques, PLIF is associated with a relatively high incidence of iatrogenic injury to musculature and neural elements (i.e., duratomy and stretching of nerve roots) due to forces from dissection and retraction [22–24]. In particular in the case presented here, PLIF carries additional risk. In the process of mobilizing the paraspinal muscles off of the patient's congenitally absent right L4 lamina, the dysmorphic thecal sack may prolapse even further, be sheered, or inadvertently be exposed to electrocautery [25]. TLIF is an alternative posterior approach that achieves discectomy and interbody fusion via a unilateral laminectomy and inferior facetectomy [26]. For this case, a TLIF could have, for the most part, avoided injury to the dysmorphic thecal sac and successfully treated the lumbar radiculopathy [24]. However, TLIF shares a similar propensity as PLIF does in terms of iatrogenic injury to paramedian muscles owing to retractile forces [27]. TLIF can also disrupt coronal balance, by nature of the single, unilaterally placed implant, and, in some instances, precipitate contralateral severe foraminal stenosis and radiculopathy, a process that theoretically may be exacerbated here given the patient's contralateral dysraphism [28]. Finally, the last posterior technique we consider is a Wiltse-approach microdiscectomy, which is a tubular, minimally invasive technique. This is a paramedian approach that achieves decompression and can be done without arthrodesis [29]. Althoughgenerally a reasonable option to treat foraminal stenosis, ultimately this microdiscectomy approach would be limited here due to ipsilateral facet hypertrophy making it technically infeasible. This approach would also expose the patient to more muscular trauma than an endoscopic transforaminal technique [30].

The anterior approaches we considered include anterior lumbar interbody fusion (ALIF) and lateral lumbar interbody fusion (LLIF). The ALIF technique places the surgeon immediately ventral to the vertebral body and disc space, facilitating decompression and fusion of the anterior spinal column without disruption of the posterior spinal tension band (i.e., posterior ligaments, paraspinal muscles, etc.) [31]. However, ALIF has a number of drawbacks for spina bifida patients in general and this patient in particular. A relative contraindication to performing ALIF is previous significant abdominal surgery [32]. This is relevant for many patients with spina bifida who may require abdominal surgery—such as ventriculoperitoneal shunt placement or treatment for urinary retention—that would theoretically make subsequent abdominal traversal for ALIF more difficult [33]. Furthermore, a challenge specific to our patient is that the level of her aortic bifurcation (although suitable lateral) at L4-5 makes it hard to achieve adequate exposure for ALIF without potentially injuring major, infrarenal blood vessels that sit ventral to the disc space [27]. LLIF is the other common anterior approach that we considered. This technique is widely employed with great success to treat degenerative spine disease via a lateral, retroperitoneal, transpsoas approach [34]. Indeed, the success of LLIF was recently highlighted in a case report of a patient with tethered cord who developed stenosis due to lumbar disc herniation at L3-4 [6]. Unfortunately, for our patient several relative contraindications for utilizing LLIF exist. For one, indirect evidence suggests that our patient's facet arthropathy and deformity (spina bifida occulta defect) would have exposed her to heightened biomechanical stress when treated with standalone LLIF (as opposed to LLIF and posterolateral fusion) [35]. Additionally, our patient's stenosis is at L4-5, a level associated with significant risk of injury to the lumbar plexus and psoas muscle when an LLIF is utilized [36].

This technical note illustrates how the unique anatomic features of spina bifida can challenge standard surgical techniques for lumbar disc herniation. Dysraphic posterior spinal column anatomy and aberrant musculature overlying the spina bifida defect may exacerbate the risk of trauma to musculature and neural elements inherent to common posterior approaches, and a history of abdominal surgery due to various spina bifida sequelae may similarly inhibit anterior techniques. We present here the first case of an endoscopic transforaminal approach to a far lateral disc in a patient with a complex spina bifida defect and degenerative changes. The technique offers a truly minimally invasive, outpatient, awake procedure that is performed through a transforaminal approach, avoiding the anatomic pitfalls that the dysraphism of spina bifida can present. At a time when adults with spina bifida are receiving surgery for degenerative disc disease at higher rates than the general population, we hope this technique will meaningfully improve the lives of patients enduring these co-occurring pathologies.
